# The feasibility of using eye‐tracking technology for cognitive screening in Down syndrome with dementia: A cross‐sectional case series

**DOI:** 10.1002/alz.70385

**Published:** 2025-06-16

**Authors:** Helen Lindner, Lotfi B. Merabet, Lars‐Olov Lundqvist

**Affiliations:** ^1^ School of Health Sciences, Faculty of Medicine and Health Örebro University Örebro Sweden; ^2^ The Laboratory for Visual Neuroplasticity, Department of Ophthalmology Massachusetts Eye and Ear, Harvard Medical School Boston Massachusetts USA; ^3^ University Health Care Research Center, Faculty of Medicine and Health Örebro University Örebro Sweden

**Keywords:** cognition, dementia, Down syndrome, eye‐tracking, screening

## Abstract

**INTRODUCTION:**

Adults with Down syndrome (DS) are at a high risk for dementia, yet cognitive screening is complicated by premorbid intellectual disabilities. This study evaluated the feasibility of using eye‐tracking technology as a screening tool.

**METHODS:**

Ten adults with DS (five with dementia, five without) completed cognitive tasks while their eye movements were recorded. Feasibility was assessed through calibration success, gaze sample quality, and task completion.

**RESULTS:**

Calibration was successful for most subjects (except one individual with dementia required five attempts and had low gaze sampling). Most subjects achieved 50%–88% gaze sample rates and completed testing with staff support. Subjects with dementia showed longer times to first fixation but similar fixation durations compared to those without dementia. Cognitive scores were lower in the dementia group but not significantly correlated with gaze quality.

**DISCUSSION:**

Eye tracking may be a feasible method for cognitive screening in DS, but further validation is needed.

**Highlights:**

- Eye‐tracking may be a potential non‐verbal method for cognitive screening in individuals with DS.
- Support from staff for engaging the subjects could be essential for maintaining attention on the computer screen.

## 1 BACKGROUND

Screening for Alzheimer's disease (AD) in individuals with Down syndrome (DS) typically involves a multimodal approach combining observational measures, neuropsychological testing, assessment of activities of daily living (ADL), and other behaviors.^1^ Observational measures are often used as initial screening for dementia in people with DS.^2^, ^3^ In Sweden, initial screening often begins when the individual with DS reaches the age of 35–40 years, using a care staff observational measure called “Early Signs.”^4^ If early indicators of dementia are detected by this measure, geriatricians and other specialists will carry out further cognitive testing and brain imaging.

In recent years, eye‐tracking technology has emerged as a promising non‐invasive method for cognitive screening, including for AD.^5^, ^6^, ^7^ Past studies have demonstrated that gaze fixation duration on target images correlated well with Mini‐Mental State Examination scores in older adults with AD.^8^, ^9^, ^10^ A recent eye‐tracking study in DS participants revealed a strong correlation between eye movements and cognitive impairments in the DS participants.^11^ However, these DS participants did not have a formal diagnosis of AD, and the 7‐s image presentation per novel image in Visual Paired Comparison (VPC) tasks might be too short for adults with DS and with slower cognitive processing speeds. While VPC tasks using novelty preference provide valuable insights into attention bias,^12^ they reflect only a limited scope of cognitive functioning.

To address these limitations, we conducted a cross‐sectional case‐series study exploring the feasibility of eye‐tracking in individuals with DS with and without a confirmed diagnosis of dementia. A series of cognitive tasks were administered to assess key eye‐tracking feasibility indicators, including successful calibration, gaze data quality, and task completion.^11^, ^13^ Given the known variability in sustained attention among individuals with DS,^14^ we also compared two commonly used eye‐tracking metrics, specifically, “gaze fixation duration” (how long they maintained their gaze on a target) and “time to first fixation” (how quickly they looked at a target) on images to examine their relevance to cognitive screening in individuals with DS.

## 2 METHODS

### 2.1 Subjects

Potential subjects were recruited from the Adult Habilitation Centre in Region Örebro County, Örebro, Sweden. Inclusion criteria were that participants had a formal diagnosis of DS or DS with dementia. Individuals with severe visual or hearing impairment that could interfere with receiving instructions and searching for images on the screen were excluded from the study. The Swedish Regional Ethical Review Board approved the study on March 10, 2021 (approval number Dnr 2021‐00874). Following ethical procedures, voluntary participation was emphasized. Both verbal and written information were provided before the subjects and/or the legal guardians gave their consent to participate.

**RESEARCH IN CONTEXT**

1. **Systematic review**: The authors reviewed the literature using traditional sources (e.g., PubMed, Scopus). Recent studies have highlighted the potential of eye‐tracking technology for cognitive screening in neurotypical adults with dementia; however, its feasibility for cognitive screening in individuals with Down syndrome (DS), both with and without dementia, has not yet been explored.
2. **Interpretation**: Our results show that DS individuals with dementia could complete the tests with some support, suggesting that eye tracking is potentially feasible to screen for cognitive changes in this group.
3. **Future directions**: Future research should focus on refining calibration procedures and developing support protocols to increase data quality. Moreover, larger screens should be used to facilitate the assessment and capture of eye‐tracking metrics in individuals with more reduced visual acuities.

### 2.2 Cognitive observational measure

Cognitive status based on the “Early Signs” measure of the DS subjects was extracted from the medical records. It is a structured questionnaire for municipal care staff to identify early signs of declining health and functioning in individuals with intellectual or developmental disabilities (ID). It covers six areas: (I) general information, (II) health, (III) functional skills, (IV) cognition and behavior, (V) background events, and (VI) summary. As for this study, we extracted the changes in scores in Area IV, which includes 13 questions (e.g., memory, orientation, language), scored by frequency (0 = not occurred to 4 = most of the day; X = not applicable).

### 2.3 Eye‐tracking based tasks for cognitive screening

**FIGURE 1:**
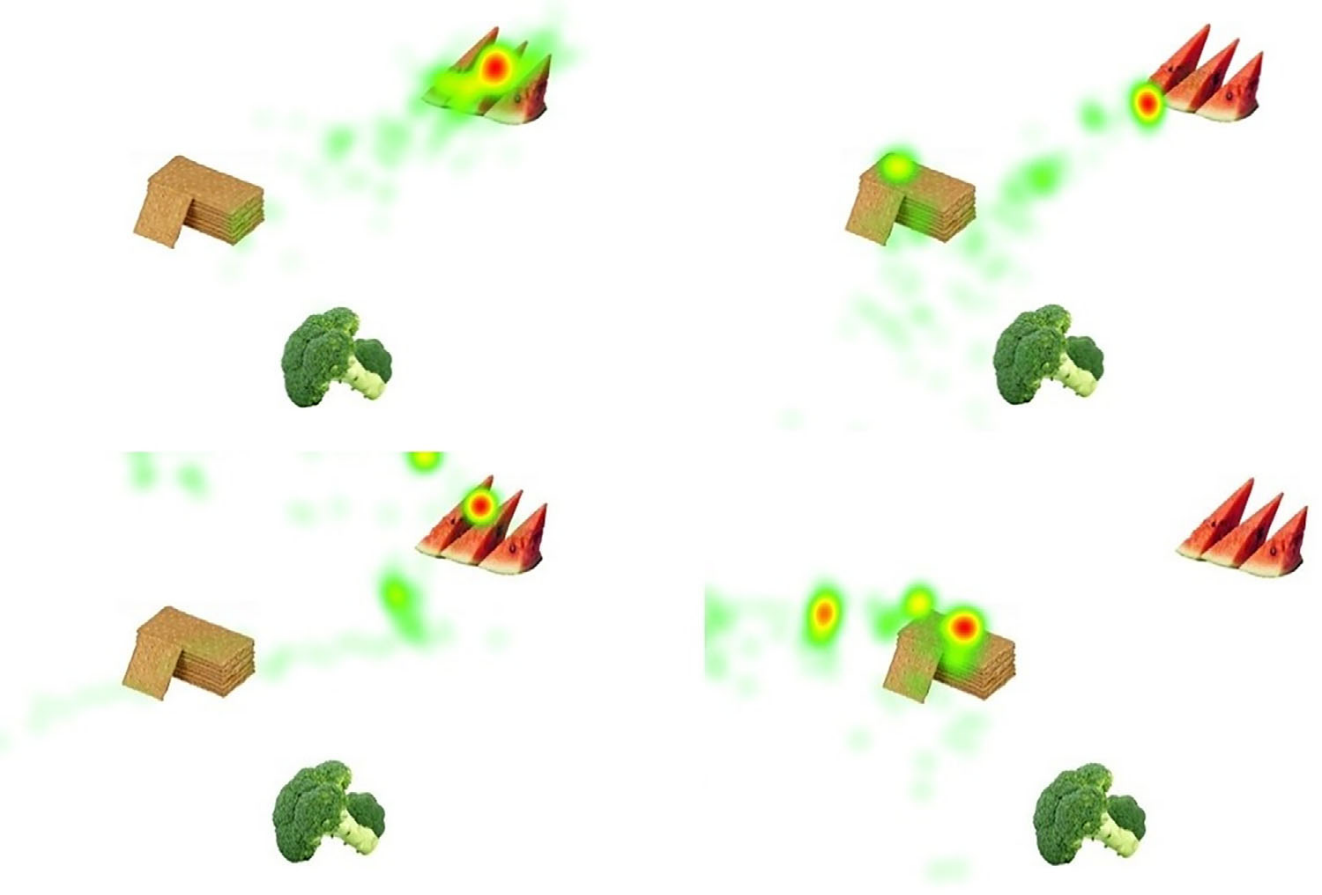
(A–D) Heatmaps of fixation durations of two subjects during a short‐term memory task. Deep red represents long fixation duration; green represents short fixation duration. Short‐term memory–Immediate recall: An image of a baby eating a watermelon was displayed. Then, three different foods were shown on the screen (watermelon, biscuits, broccoli). The subject was asked, “Do you remember what the baby was eating?” A (top left): a Down syndrome subject without dementia; B (top right): a Down syndrome subject with dementia. Short‐term memory–Delayed recall (after 5 min): The same three foods at the same location were shown on the screen (watermelon, biscuits, broccoli). The subject was asked again, “Do you remember what the baby was eating?” C (bottom left): a Down syndrome subject without dementia; D (bottom right)—one Down syndrome subject with dementia.

Three occupational therapists (including the first author) at the Adult Habilitation Center, with 5–20 years of clinical experience working with patients with DS and ID, selected cognitive tasks based on two criteria: (a) their relevance to the everyday lives of individuals with DS or ID, and (b) tasks that can be answered by recording eye gaze responses. The tasks are listed below (see Figure 1 for visualizations of the recall tasks).

#### 2.3.1 Sustained attention

Observing and tracking a car moving at a speed of 0.54 s from right to left (total video time of 30 s). The participant was prompted to track the moving car until it disappeared from the screen. The task was shown twice: once at the beginning and once at the end of the assessment. This was to assess possible attentional fatigue and decline by the end of the test.

#### 2.3.2 Short‐term memory

1. Immediate recall: An image of a baby eating a watermelon was displayed. Then, three different foods were shown on the screen (watermelon, biscuits, broccoli). The participant was asked, “Do you remember what the baby was eating?”
2. Delayed recall (after 5 min): The same three foods at the same location were shown on the screen (watermelon, biscuits, broccoli). The participant was asked again, “Do you remember what the baby was eating?”

#### 2.3.3 Orientation

1. To time: An image of the sun and moon was shown on the screen. The participant was prompted, “Look at the sun if it is daytime and look at the moon if it is nighttime.” (All tests were performed during daytime.)
2. To space: An image of a computer and a bus were shown on the screen. The participant was prompted, “Look at the computer if you were sitting in front of the computer and look at the bus if you were sitting inside the bus.” (The correct answer was always sitting in front of the computer.)
3. To direction: Two images of two apples were shown at the same time (one on the left and one on the right, as well as one at the top and one at the bottom). The participant was prompted, “Look at the apple on the right, the one on the left, the one at the top, and the one at the bottom.”

#### 2.3.4 Discrimination

1. Sound
  - A dog and a cat were shown on the screen. Then, a dog barking sound was played. The participant was prompted, “Look at the correct image associated with the sound.”
  - A car and a person washing their hands under a running tap are shown on the screen. Then, the sound of running tap water was played. The participant was prompted, “Look at the correct image associated with the sound.”
2. Size and form
  Three sets of images are shown:
  - An image with three triangles of different sizes. The participant was prompted, “Look at the smallest triangle.”
  - An image with three circles of different sizes. The participant was prompted, “Look at the largest circle.”
  - An image with six triangles of different sizes and only one small circle. The participant was prompted, “Look at the circle.”

The task images were placed on a widely spaced grid, following Wilkinson's recommendations for arranging visual displays in augmentative and alternative communication for individuals with DS.^15^, ^16^ The screen was divided into a 3 × 3 grid, and one image occupied one cell of the grid.

### 2.4 Collection of eye‐tracking data

A Tobii Pro fusion eye tracker (sampling rate 250 Hz, Tobii Sweden) was used to record eye movements during testing. The eye tracker was mounted on the lower edge of a 15.6‐inch laptop screen with a display resolution of 1920 × 1080 pixels. All subjects were seated at a viewing distance of approximately 50 cm from the screen.

Calibration was performed using the built‐in two‐point target calibration procedure (positioned to the left and right on the screen). After successful calibration, the test images and videos were displayed using the Tobii Pro Lab (1.217 version) software. The first author (H.L.) read each question and allowed the subject to respond by looking at the correct image on the screen. The subject tapped the table or said “yes” when they were ready for the next question. The same set of images for each task was shown twice: once when the question was read and once for the subject to respond. Unlike other eye‐tracking tests for AD that impose time limits for responses,^8^, ^9^, ^11^ we decided to allow enough time for subjects to answer, given that the aim was to investigate cognitive functions.

### 2.5 Feasibility metrics

First, we investigated feasibility using the following three eye‐tracking metrics according to Dunn et al.^17^ and Piro‐Gambetti^11^: successful calibration, gaze data quality, and task completion rate. *Successful calibration* was defined as accurate alignment of the eye tracker with the subject's gaze, as determined by the Tobii Pro fusion calibration criteria, to ensure precise detection of eye movements. This involved the subject correctly focusing on the two calibration points, allowing the eye tracker to map their eye positions reliably before starting the screening test. *Gaze data quality* was the percentage of valid gaze samples captured by the eye tracker during cognitive tasks. As suggested by Piro‐Gambetti,^11^ 50% gaze samples are adequate for individuals with DS. *Task completion rate* is the proportion of cognitive tasks successfully completed by each subject within the whole test (defined as producing valid gaze data without the need to terminate prematurely). Second, we investigated feasibility based on the support by care staff and the ability to tap the table or said “yes” when they were ready for the next question.

### 2.6 Data analysis

The eye‐tracking recordings were first inspected for sudden head movements that could affect gaze fixations. Using the Area of Interest (AOI) tool of Pro Lab, the moving car and the correct image targets were manually drawn to cover the cell where the image was located. Heat maps were also generated to visualize total fixation duration for each cognitive task. For each correct/target image, we extracted the “time to first fixation” and the “total fixation duration” for each subject.

To calculate a cognitive score using eye‐tracking data, we employed the same method reported by Oyama et al.^10^ We used the total fixation duration on the AOI (corrected answer) and the total fixation duration on the whole screen for each task. A percentage for each task was then calculated using the following formula:

$$\begin{array}{c} \mathrm{Eye}\;\mathrm{tracking}\;\mathrm{based}\;\mathrm{cognitive}\;\mathrm{score}\;\left(\mathrm{total}\;\mathrm{fixation}\;\mathrm{duration}\right)\\ =\frac{\mathrm{total}\;\mathrm{fixation}\;\mathrm{duration}\;\mathrm{on}\;\mathrm{the}\;\mathrm{AOI}}{\mathrm{total}\;\mathrm{fixation}\;\mathrm{duration}\;\mathrm{on}\;\mathrm{the}\;\mathrm{whole}\;\mathrm{screen}\;}\;\;X\;100\% \end{array}$$

The task percentages were then averaged for each subject to produce a cognitive score.

## 3 RESULTS

### 3.1 Timing of diagnosis, mean age, and intellectual disability

All DS subjects had a DS diagnosis according to International Classification of Diseases (ICD) Q90.9 criteria. Five of them received the diagnosis of dementia at least 1 year prior to testing. The DS group with dementia (*n* = 5, three male, two female) had a mean age (51.6 years ± 3.36 SD), which is slight higher than the DS group without dementia (48.2 years ± 10.1 SD). Both DS groups each had one subject with mild ID and the rest of the subjects had moderate ID.

### 3.2 Early Signs measure

In general, the DS subjects with dementia showed an increase in difficulty in the area “cognition” (mean score 41) as compared to the DS subjects without dementia (mean score 25). In the two questions regarding problems with memory and concentration, all DS subjects with dementia scored *3‐several times a month t*o *4‐several times per day* over a period of 5 months. In the question “have problem to remember names of different objects”, Subjects C and E were scored *3‐several times a month to 4‐several times per day* over a period of 5 months. In the question “ask the same question again and again,” subject D scored 3‐several times a month to 4‐several times per day over a period of 5 months. For DS subjects without dementia, all the areas were *0‐not observed or 1‐happen several times per month* over a period of 5 months.

### 3.3 Feasibility metrics

Table 1 presents the metrics that show the feasibility of eye tracking for DS subjects. One subject in the DS group with dementia required five attempts for successful calibration. The gaze sample of this subject was only 12%, whereas the gaze sample of the rest of the subjects ranged from 50% to 88%.

**TABLE 1:** Feasibility metrics

Parameter	Age	Intellectual disability	Calibration—(number of attempts)	Gaze sample collected during the test (%)	Task completion (Yes/No), completion time (seconds)	Need for a staff member to sit beside subject during eye tracking test	Able to indicate they had answered the question/task
DS with dementia							
A	57	Moderate	5	12	Yes, 455,71	Yes	Yes
B	49	Moderate	2	51	Yes, 415.68	Yes	Yes
C	49	Moderate	3	12	Yes, 600.85	Yes	Yes
D	52	Mild	1	89	Yes, 495.67	No	Yes
E	50	Moderate	1	50	Yes, 444.01	No	Yes
DS without dementia							
F	63	Moderate	3	63	Yes, 377.87	Yes	Yes
G	35	Moderate	1	64	Questionable, 515.83	Yes	Yes
H	45	Moderate	2	77	Yes, 554.82	No	Yes
I	51	Moderate	1	51	Yes, 505.17	No	Yes
J	48	Mild	1	88	Yes, 302.21	Yes	Yes

Abbreviation: DS, Down syndrome.

All subjects (except subject G without dementia) were able to complete the whole test based on the gaze measurements of the last task “moving car video” (see Table 2, sustained attention, the end). Five subjects required a staff member to sit next to the subject during testing. This was to help with maintaining attention on the screen. All subjects were able to indicate they had answered the question/task.

**TABLE 2:** Individual task scores and mean cognitive scores

	Sustained attention (moving car video %)		Short term memory (% correct)		Orientation (% correct)			Discrimination (% correct)		
Subject	The Beginning	The End	Recall	Delayed recall	Time	Space	Direction Left/right. Up/down	Size and form Triangles, circles	Sound Water and dog	Cognitive score (average of all tasks%)
DS with dementia										
A	33.00	0.53	0.00	0.00	19.55	48.09	94.44, 0.00 9.39, 0.00	0.29, 83.57, 10.00	29.25, 0.00	21.87
B	26.32	10.18	12.27	10.43	30.44	20.48	1.99, 0.00 0.89, 33.14	0.00, 0.59, 0.00	54.11,19.62	14.70
C	71.00	8.14	0.00	4.80	61.36	0.00	0.00, 0.00 31.69, 10.21	0.00, 0.00, 0.00	0.00, 0.00	12.48
D	57.64	70.28	4.85	4.89	0.00	34.80	1.23, 7.71 70.34, 18.64	2.22, 72.91, 2.31	66.83, 10.02	28.31
E	0.17	0.97	0.53	3.58	0.00	4.14	0.00, 33.08 0.00, 0.00	0.00, 0.00, 0.00	0.00, 0.00	2.83
Mean cognitive score (subject A to E) = 16.04										
DS without dementia										
F	21.00	13.53	16.47	0.00	18.65	5.21	1.20, 0.34, 0.00, 17.26	0.20, 0.00, 0.00	78.18, 32.36	13.63
G	8.00	0.00	2.32	12.74	0.64	44.44	0.11, 34.22 0.19, 26.33	0.00, 0.00, 0.00	11.85, 5.92	9.78
H	87.16	70.57	46.61	64.95	47.66	75.08	12.68, 1.74. 68.27, 7.16	50.11, 17.03, 30.52	1.01, 95.45	45.07
I	40.68	36.72	27.61	14.62	25.02	15.29	21.61, 0.35 38.13, 19.20	10.16, 58.36, 3.94	45.16, 46.00	26.86
J	88.00	74.47	13.19	42.74	15.27	15.93	8.84, 11.06 21.07, 52.80	37.73, 60.68, 47.39	15.03, 24.52	35.25
Mean cognitive score (Subject F to J) = 26.12										

Abbreviation: DS, Down syndrome.

### 3.4 Time to first fixation on target images

For the five subjects with dementia, the average time to first fixation on target images (correct answer) was 3.22 s (± 3.93 SD). For the five subjects without dementia, the average time to first fixation on target images was 2.28 s (± 3.36 SD).

### 3.5 Fixation duration on target images

For the five subjects with dementia, the average fixation duration on target images was 2.58 s (± 4.31SD). For the five subjects without dementia, the average fixation duration on target images was 2.65 s (± 4.65SD).

### 3.6 Relationship between cognitive scores and eye‐tracking performance

Table 2 presents the cognitive scores and individual task scores for each subject. For the five subjects with dementia, the average cognitive score was 16.04. For the five subjects without dementia, the average cognitive score was 26.12. The Pearson correlation between cognitive scores and gaze samples was 0.20 (*p* = 0.58), whereas the Pearson correlation between cognitive scores and task completion time was 0.11 (*p* = 0.77).

## 4 DISCUSSION

The findings of this feasibility study provide preliminary support for the use of eye‐tracking technology in individuals with DS with and without dementia. Although calibration was successful for all subjects, one subject without dementia required five attempts and showed notably low gaze sampling (12%), indicating potential challenges in this population. In contrast, the remaining subjects had acceptable gaze sampling rates ranging from 50% to 88%, which was similar to the recent study with the same population.^11^

Despite variability in gaze quality, nearly all subjects were able to complete the testing protocol. This suggests that the procedure, including staff support for maintaining attention, was effective in facilitating task completion. Indeed, five participants required the presence of a staff member to help maintain attention, highlighting the importance of support when implementing eye tracking in populations with DS and ID.

Subjects with and without dementia demonstrated comparable performance in fixation duration on target images, suggesting that once attention was directed to the correct stimuli, the ability to sustain visual attention did not differ substantially between groups. However, a group difference was observed in the time to first fixation on target images, with the dementia group showing longer and more variable times. This may reflect differences in attentional control or visual search strategies associated with cognitive decline.

Similar to the previous findings,^8^, ^9^, ^10^ our cognitive scores further support the presence of cognitive differences between the groups, with lower average scores in the dementia group. Both DS groups included one subject with mild ID and these two subjects showed the highest cognitive scores within their own group. This may suggest that severity of ID may influence the cognitive score. However, the modest and non‐significant Pearson correlation between cognitive scores, gaze samples, and task completion suggests that gaze behavior may only partially reflect cognitive ability in this group.

Overall, the results demonstrate that eye tracking could be a feasible method for assessing cognitive functions in DS individuals with dementia, if staff support was given. Future research should focus on refining calibration procedures and developing support protocols to increase data quality.

## CONFLICT OF INTEREST STATEMENT

The authors declare no conflicts of interest. Author disclosures are available in the supporting information.

## Supporting information



Supporting Information
